# Hypervirulent *Klebsiella pneumoniae* as Unexpected Cause of Fatal Outbreak in Captive Marmosets, Brazil 

**DOI:** 10.3201/eid2612.191562

**Published:** 2020-12

**Authors:** Juliana Mariotti Guerra, Natália Coehlo Couto de A. Fernandes, Alessandra Loureiro Morales dos Santos, Joana de Souza Pereira Barrel, Bruno Simões Sergio Petri, Liliane Milanelo, Monique Ribeiro Tiba-Casas, Alcina Maria Liserre, Cláudia Regina Gonçalves, Cláudio Tavares Sacchi, José Luiz Catão-Dias, Carlos Henrique Camargo

**Affiliations:** Instituto Adolfo Lutz, São Paulo, Brazil (J.M. Guerra, N.C.C.A. Fernandes, A.L.M. dos Santos, J.S.P. Barrel, M.R. Tiba-Casas, A.M. Liserre, C.R Gonçalves., C.T Sacchi., C.H. Camargo);; Universidade de São Paulo, São Paulo (N.C.C.A. Fernandes, A.L.M. dos Santos, J.L. Catão-Dias);; Parque Ecológico do Tietê, São Paulo (B.S.S. Petri, L. Milanelo)

**Keywords:** bacteria, disease outbreaks, epizootic, Gram-negative bacteria, Klebsiella pneumoniae, One Health, marmosets, primate diseases, pulsed-field gel electrophoresis, South America, zoonoses

## Abstract

After the sudden death of captive marmosets in São Paulo, Brazil, we conducted a histologic and microbiologic study. We found hyperacute septicemia caused by hypermucoviscous sequence type 86 K2 *Klebsiella pneumoniae.* We implemented prophylactic antimicrobial therapy, selected dedicated staff for marmoset interactions, and sanitized the animals’ fruit to successfully control this outbreak.

*Klebsiella pneumoniae* is an opportunistic bacteria that is a normal part of the nasopharyngeal and gastrointestinal tract microbiome of humans and animals (*1*). The hypermucoviscous variant of *K. pneumoniae* (hvKp), initially described in Southeast Asia, has emerged as a pathogen affecting young and healthy persons worldwide (*2*). The development of prominent polysaccharide capsules associated with capsular serotypes K1 or K2 have been reported as the major virulence determinants for human hvKp in liver abscesses, perhaps because it seems to protect the bacteria from phagocytosis and prevents destruction by bactericidal serum factors (*2**)*. 

*K. pneumoniae* strains have also been associated with a variety of diseases in animals, especially in Old World (Africa, Asia, and Europe) and New World (Oceana, North America, and South America) nonhuman primates (*3**–**5*). Sudden death or various clinical signs, including anorexia, prostration, fever, cough, dyspnea, mucopurulent discharge, meningitis, pneumonia, peritonitis, and sepsis are strongly associated with sporadic infections of *K. pneumoniae* in common marmosets research colonies (*5*,*6*). 

Despite the well-recognized zoonotic importance of hvKp and the public health risk of emerging multidrug-resistant strains (*7**–**9*), information is incomplete about the genotypic and phenotypic characterization of the etiologic agent essential to adequately diagnose and treat this pathogen in captive and wild nonhuman primates. The aim of this study was to report an epizootic among common marmosets in a wildlife rehabilitation center in Brazil and to describe the serotype, sequence typing, virulence properties, and resistance profile of the *K. pneumoniae* strains involved. 

## The Study

On February 10–11, 2019, a total of 11 captive marmosets (8 *Callithrix penicillata*, 2 C. *jacchus*, and 1 hybrid) died suddenly without clinical signs of disease. All of the animals were maintained in Parque Ecológico do Tietê, located in São Paulo municipality, São Paulo, Brazil, which is a center for receiving, rehabilitating, and referring wildlife. All animals had been in captivity 123–399 days. No new animals had been introduced into the cages in the previous 25 days. Each necropsy was performed <24 h after death in accordance with the Brazil Ministry of Health’s guide for surveillance of epizootics in nonhuman primates (*10*). Tissue samples were preserved in phosphate-buffered formalin 10% and processed for routine histopathology and for 12 hours in refrigeration for microbiologic and molecular analyses. This study was approved by the Ethics Committee for the Use of Animals (CEUA) of Adolfo Lutz Institute, Brazil (protocol no. 11/2016), SISBIO registration no. 50551 for the manipulation of wildlife material, and SISGEN registration nos. A1A2A72 and A7EB4B6. 

**Figure 1:**
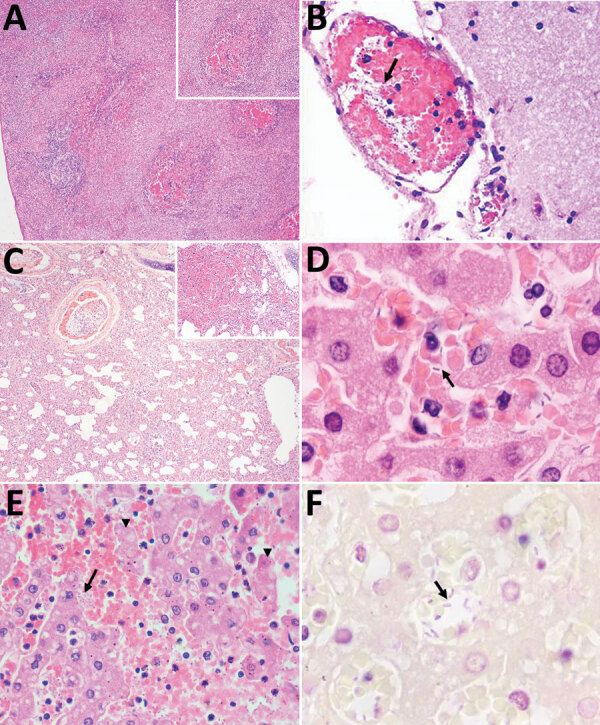
Microscopic findings of histological and histochemical examination of tissue samples from captive marmosets in investigation of a fatal epizootic caused by highly virulent *Klebsiella pneumoniae* sequence type 86 strain P04 in Brazil, 2019. A) Spleen shows necrosis in germinal centers, suppurative splenitis, and hemorrhage (inset: necrosis in germinal center). Hematoxylin and eosin stain (H&E); original magnification ×4. B) Brain (meninges) shows bacterial rods inside vascular lumen (arrow). H&E stain; original magnification ×40. C) Lung shows sinterstitial pneumonia (H&E stain; original magnification ×4) and alveolar hemorrhage (inset; H&E stain; original magnification ×10). D–F) Liver samples. D) Numerous intravascular bacilli (arrow). H&E stain; original magnification ×100. E) Hepatocellular necrosis (arrowheads) associated with numerous bacterial rods (arrow) and neutrophils in the sinusoids. H&E stain; original magnification ×40. F) Sinusoids filled with gram-negative bacterial structures (arrow) and neutrophils. Gram stain; original magnification ×1,000.

Histologic findings from all of the animals were compatible with hyperacute septicemia. Multiple sections of liver, spleen, and adrenal tissue were similarly affected by suppurative and necrotizing multifocal lesions associated with intrahistiocytic gram-negative bacteria, 1–2-μm long. We also observed numerous intravascular gram-negative bacilli in samples from the liver (10 of 10 samples; the sample from 1 marmoset was excluded because the animal showed severe postmortem autolysis), cerebrum (8/10), lungs (3/10), heart (1/7), intestines (1/7), thymus (1/2), and skeletal muscle (1/1). Other relevant microscopic findings from different tissues are summarized in Table 1 and Figure 1. We found no microscopic alterations in the analyzed fragment samples from the stomach, tongue, testis, thymus, skin, uterus, or lymph nodes. 

**Table 1:** Histologic findings for tissue samples from captive marmosets analyzed by microscopic evaluation in investigation of a fatal epizootic caused by highly virulent *Klebsiella pneumoniae* sequence type 86 strain P04 in Brazil, 2019

Organ	No. samples	Histologic findings	Positive/total
Liver	10	Sinusoidal leukocytosis, predominantly with neutrophilia	9/10
		Hemorrhagic foci	7/10
		Hepatitis necrotizing, suppurative, acute, multifocal	2/10
		Intravascular fibrin deposition	1/10
Spleen	9	Splenitis necrotizing, suppurative, acute, multifocal	8/9
		Hemorrhage	8/9
		Many bacilli on the red pulp	8/9
Lung	10	Subacute interstitial pneumonia	8/10
		Occasional free bacilli	7/10
		Hemorrhage	1/10
Cerebrum	10	Bacilli on leptomeninges	1/10
Adrenal	2	Adrenalitis necrotizing, suppurative, acute, multifocal	2/2
Heart	7	Myocarditis necrotizing, acute, multifocal	1/7
Intestine	7	Enteritis neutrophilic, acute, diffuse	2/7
Kidney	8	Granular tubular casts	4/8
		Tubular acute necrosis	1/8

**Figure 2:**
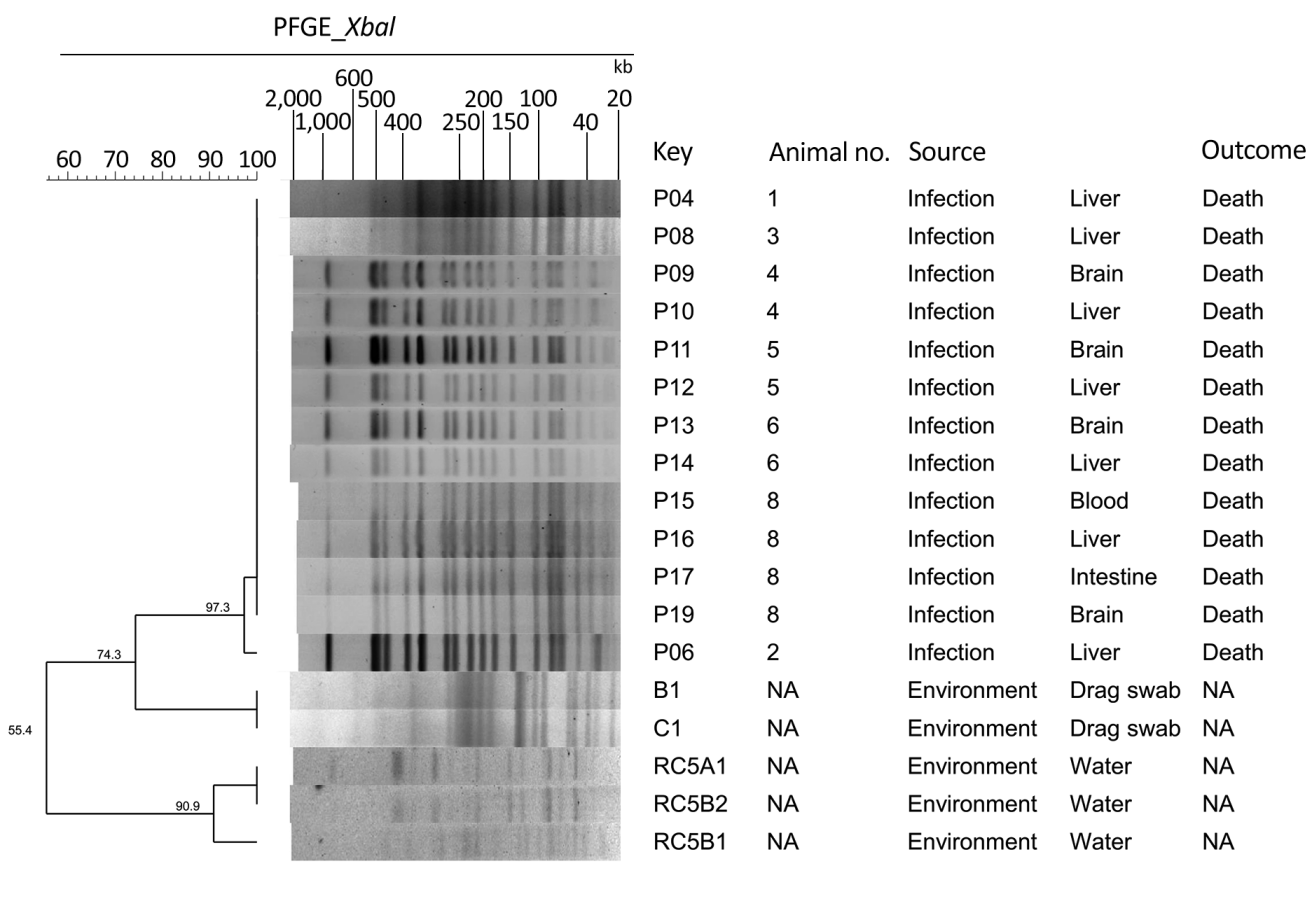
Dendrogram and pulsed-field gel electrophoresis (PFGE) typing of XbaI-restricted *Klebsiella pneumoniae* strains from captive marmosets in investigation of a fatal epizootic caused by highly virulent *K. pneumoniae* sequence type 86 in Brazil, 2019. PFGE profiles were defined based on 100% Dice similarity cutoff value of the UPGMA clustering method (1.5% optimization; 1.5% tolerance). The Universal Size Standard Strain H9812 (*Salmonella* Braenderup) was used as reference in all gels. NA, not applicable.

Pure cultures of *K. pneumoniae,* positive for the string test (*11*), were recovered from the brain and liver in 8 different animals. The representative isolate P04 displayed susceptibility to all the antimicrobial agents we evaluated using the broth microdilution methodology with Sensititre plate (Thermo Fisher Scientific, https://www.thermofisher.com), according to the manufacturer’s instructions (Table 2). We screened for *K. pneumoniae* in environmental samples of water from a lake near the primate cages by filtration and in drag swabs from their cages by direct growth on MacConkey agar plates. Although we recovered *K. pneumoniae* isolates from both samples, all of them were negative in the string test. We subjected all isolates identified as *K. pneumoniae* to DNA macro-restriction by using 30 U of XbaI enzyme followed by pulsed-field gel electrophoresis (PFGE) (https://www.cdc.gov/pulsenet/pathogens/pfge.html). PFGE results showed the same restriction profile among the isolates recovered from the dead animals; however, environmental isolates clustered apart from the invasive isolates (Figure 2).

**Table 2:** Antimicrobial susceptibility profile of highly virulent *Klebsiella pneumoniae* sequence type 86 strain P04 from a fatal epizootic among captive marmosets in Brazil, 2019*

Antimicrobial	MIC, mg/L†	Category
Amikacin	<4.0	Susceptible
Ampicillin/sulbactam	8/4	Susceptible
Aztreonam	<2	Susceptible
Cefepime	<2	Susceptible
Cefotaxime	<2	Susceptible
Ceftazidime	<1.0	Susceptible
Ciprofloxacin	<0.06	Susceptible
Colistin	<0.25	Susceptible
Doripenem	<0.5	Susceptible
Doxycycline	2.0	Susceptible
Gentamicin	<1.0	Susceptible
Imipenem	<1.0	Susceptible
Levofloxacin	<1.0	Susceptible
Meropenem	<1.0	Susceptible
Minocycline	4.0	Susceptible
Piperacillin/tazobactam	<8/4	Susceptible
Polymyxin B	<0.25	Susceptible
Sulfamethoxazole/trimethoprim	<0.5/9.5	Susceptible
Ticarcillin/clavulanic Acid	<16/2	Susceptible
Tigecycline‡	0.5	Susceptible
Tobramycin	<1.0	Susceptible

*Susceptibility determined by Sensititre (ThermoFisher, https://www.thermofisher.com). †MIC values were categorized as susceptible, intermediate, or resistance following Clinical and Laboratory Standards Institute (https://clsi.org) M100-S30 breakpoints (http://em100.edaptivedocs.net/dashboard.aspx). ‡Tigecycline breakpoint followed Food and Drug Administration recommendations.

We subjected the P04 strain to whole genome sequencing using the Thermo Fisher Ion Torrent S5 platform, resulting in 1,049,163 reads; the de novo assembled genome comprised 5,358,608 bps grouped in 62 contigs, with an average coverage depth of 53. The whole-genome shotgun project reported here was deposited in DDBJ/EMBL/GenBank under accession number SPSP00000000. 

We employed online platforms from PubMLST (https://pubmlst.org) to definitively identify species as *K. pneumoniae*, sequence type (ST) 86, capsular type K2, and multiple virulence genes: *mrkABCDFHIJ* cluster (mannose-resistant *Klebsiella*-like type III fimbriae cluster, associated with adhesiveness and fimbrial filament formation to adhere to eukaryotic cells) (*12*) in the same contig of the *kvgAS* genes; *iroBCD* genes (salmochelin) in the same contig with the *rmpA* gene (regulator of mucoid phenotype A); and *rmpA2* gene within the contig along the *iucABD* with the *iutA* genes (aerobactin) (http://bigsdb.pasteur.fr). We detected only the constitutive antimicrobial-resistant genes *bla*_SHV-1_, *oqx*AB, and *fos*A by the in silico analysis (Comprehensive Antibiotic Resistance Database, https://card.mcmaster.ca). Phylogenetic analysis of high quality single-nucleotide polymorphisms (SNPs) built on the CSI Phylogeny 1.4 (Center for Genomic Epidemiology, https://cge.cbs.dtu.dk/services/CSIPhylogeny) showed that the ST86 isolates were closely related and the P04 strain clustered closely with IPEUC340, an isolate recovered in 1975 in France (Appendix). The isolate RJF293, ST374, clustered apart from the ST86 isolates (Appendix Figure). 

To contain the spread of hvKp to other animals, metaphylatic therapy with trimethoprim/sulfamethoxazole was implemented by adding the antimicrobial to the water supply of animals during the first 5 days after the fatal cases were identified. In addition, access to the cages was restricted to a dedicated employee, who wore clothing exclusively for accessing the cages for the duration of the outbreak. We also implemented additional steps for sanitizing fruit eaten by the marmosets with 2% sodium hypochlorite for 15 minutes and dedicated space and staff for preparing the marmosets’ meals after the epizootic event. 

## Conclusions 

We report the detection of hypermucoviscous *K. pneumoniae* ST86 K2 as cause of a sudden fatal outbreak in captive marmosets. Implementing prompt containment measures led to successful control of this outbreak. The burden of hypermucoviscous *K. pneumoniae* ST86 K2 in unexpected reservoirs, including those in contact with people, deserves further investigation. The emergence of these strains is a concern for human and veterinary health because of the potential for these bacteria to acquire multidrug-resistant genes, their capacity to persist in the environment and to infect a wide range of hosts, and the unavailability of vaccines against these strains for humans and animals (*13*). The expansion of this emerging pathogen among different reservoirs should be carefully surveilled, because the relationship between hypervirulent and multidrug-resistant strains is narrowing. 

Appendix Additional information for study of hypervirulent *Klebsiella pneumoniae* as unexpected cause of fatal outbreak in captive marmosets, Brazil.
