# Supplementary material for: Hypervirulent Klebsiella pneumoniae as Unexpected Cause of Fatal Outbreak in Captive Marmosets, Brazil
Source: Emerg Infect Dis. 2020 Dec;26(12):3039–43. doi: 10.3201/eid2612.191562 (PMC7706955; doi:10.3201/eid2612.191562)
Supplement: Appendix — Additional information for study of hypervirulent Klebsiella pneumoniae as unexpected cause of fatal outbreak in captive marmosets, Brazil. [file 19-1562-Techapp-s1.pdf]

Article DOI: https://doi.org/10.3201/eid2612.191562

Hypervirulent Klebsiella pneumoniae as Unexpected Cause of Fatal Outbreak in Captive Marmosets, Brazil

Appendix

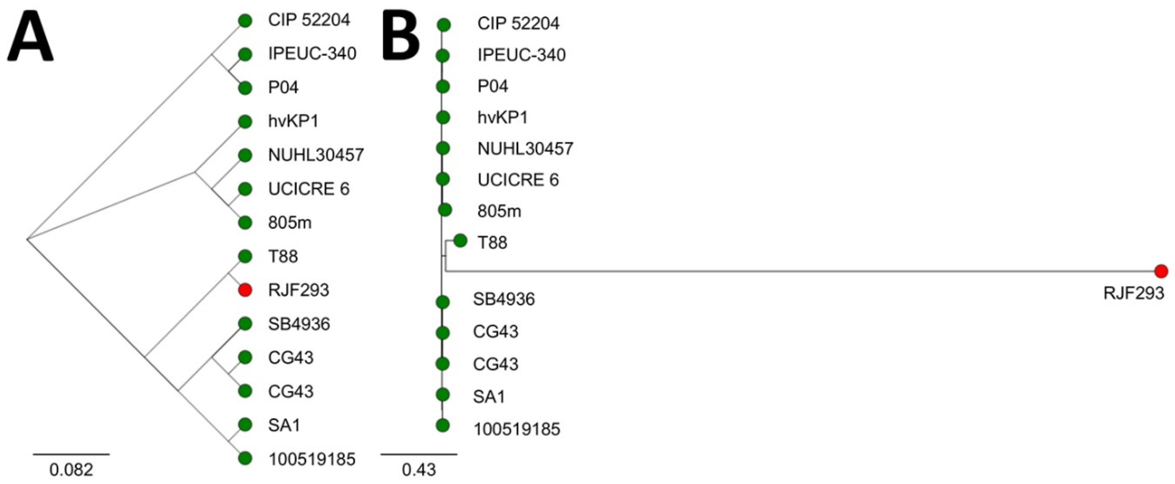

Appendix Figure. Schematic representations of high quality SNPs trees built from publicly available ST86 *K. pneumoniae* genomes. In A) it is possible to note that the P04 isolate described in this study clustered close to the IPEUC-340 isolate, recovered in 1975 in France. In B) we observe the high similarity of ST86 *K. pneumoniae* genomes in comparison with the outgroup strain RJF293, an ST374 hypermucoviscous *K. pneumoniae* recovered from invasive infection. Green dots represent ST86 isolates; red dot represents ST374 isolate.
